# Illustrations of the Elementary Forms of Disease

**Published:** 1836-10

**Authors:** 


### Art. VII.

*Illustrations of the Elementary Forms of Disease.* By Robert Carswell, m.d., Professor of Pathological Anatomy in the University of London, &c. Fasciculus IX. and X.—*London,* 1836. Fol. Pp. 34. Eight Plates.

These two numbers of Dr. Carswell's classical work are devoted to Hypertrophy and Atrophy. The application of the term Hypertrophy (one of comparatively recent introduction,) has by no means been limited to the class of changes to which it was at first applied, such as to increased thickness of the walls of the heart, &c. M. Andral, for instance, regards the grey or semitransparent striæ of cancer, or the dead white substance of encephaloid tumours, as depending on a change similar to that by which the muscles of the arm increase by exercise; the one being hypertrophy of the cellular membrane, the other hypertrophy of the muscular tissue. He thus classes together two changes of the most opposite character, except in one particular, the appearance of the parts on dissection. No practical deductions could be drawn from this arrangement, even if mischief did not arise from it; and this alone warrants the suspicion that it is an instance of hasty generalization. The error has arisen from not keeping in view the necessity of regarding all morbid changes in connexion with their symptoms. "One of the advantages," says Dr. Baillie, "arising from the more attentive examination of morbid structure is, that we shall be able to distinguish between changes which may have some considerable resemblance to each other, and which have been generally confounded. This will ultimately lead to a more attentive observation of symptoms while diseased actions are taking place, and be the means of distinguishing more accurately diseases. When this has been done, it will be more likely to produce a successful enquiry after a proper method of treatment." An exclusive attention to appearances alone, without regarding the history of the disease, lead M. Andral to confound together diseases which should be divided, and he has consequently missed the end to which every branch of medicine should tend, the cure of disease.

Dr. Carswell has taken a more philosophical view of the question, and, from an examination of the causes of those forms of hypertrophy which are the best defined, he has reduced it to more narrow limits, by showing that it should be regarded as a physiological act. The grounds of his conclusion are, that hypertrophy, or an increase of the healthy structure of an organ, depends on an increase of its healthy function: the muscular arms of the blacksmith and the legs of the opera dancer prove this, as well as the increased size of the muscular fibres of the bladder, heart, &c., after they have had for a length of time to overcome some mechanical obstacle to the free passage of their contents. For increased function a larger supply of blood is necessary, which is proved by the enlarged state of the vessels. Thus, when one kidney is wasted, and the other proportionably enlarged, its vessels undergo a corresponding increase in size. Hypertrophy is therefore a condition precisely similar to that required for the general increase of the whole body; and it is fallacious to suppose that a healthy act can lay the foundation for a product, like carcinoma, altogether foreign to the constitution. This limitation of hypertrophy to a physiological act, which only becomes pathological when it interferes with the due performance of an organ, appears to be unobjectionable; and the practical advantage resulting from it is, that it involves the principle on which the successful treatment of hypertrophy is founded.

Dr. Carswell classes the varieties of hypertrophy according to their probable causes. These are either general or local. The most remarkable of the former seems to consist in a predisposition or peculiar organization of the individual, by which nutrition in general is so modified as to give rise to an increased but imperfect development of some tissues or organs. Such is the case in scrofulous persons, in whom the liver, bones, lymphatic glands, sometimes the brain, the upper lip, &c., are enlarged. The excessive deposition of fat is another instance. The local causes are divided into three. The first cause is the increased action of an organ in the healthy exercise of its function. The most obvious example is in the voluntary muscles, particularly of the limbs, which, though not constituting a pathological condition, cannot exist to a great extent in one region of the body without exercising a marked influence in the development of some other region, according to the law of regular organic development. Thus, the legs of a drayman seem too slender to support his broad chest and brawny shoulders. An important variety consists of great hypertrophy of the heart without any perceptible mechanical obstacle, or local morbid stimulus, that can be traced; the excess of nutrition depending on an increased exercise of the heart, induced, in a manner not satisfactorily explained, by certain diseased states of other organs, particularly of the brain and nervous system, and various affections of the mind. The second local cause of hypertrophy is a mechanical obstacle to the function of an organ, which includes a large number of well-known instances; as hypertrophy of the heart from diseased valves, of the stomach from contraction of the pylorus, &c. The third cause of hypertrophy is the long-continued influence of a morbid stimulus. This is considered by Dr. Carswell as the most frequent local cause, and is seen in hypertrophy of the mucous, cutaneous, cellular, fibrous, and osseous tissues, and glandular organs, produced by a state of irritation or chronic inflammation.

Dr. C. insists on the necessity of a minute examination, to prevent confounding with true hypertrophy an increase of bulk from the deposition of lymph; and adds, that the physical characters of coagulable lymph effused and organized in the cellular tissue, and on serous membranes, are so similar to the original structures, that in most cases it is impossible to ascertain whether the increase of bulk is owing to hypertrophy or to the presence of an analogous new formation. In the instances which are given, we do not think that Dr. C. has kept within the limits of his definition. No instances can be admitted except those in which chronic inflammation has acted as a stimulus, by which a greater quantity of blood has been attracted to the part. If inflammation has been set up in any organ, and the inflamed part is increased in bulk, that change cannot be attributed to hypertrophy; as the first is a new, a morbid action; and the second is, according to Dr. C., merely an increase of the healthy nutritive function of a part. Wherever, therefore, symptoms of inflammation had existed during life, and, on dissection, a thickened state of the parts is found, we conceive it would be more convenient and correct to distinguish such changes from hypertrophy. The impossibility in many cases, which Dr. C. admits, of distinguishing between organized lymph and the proper tissue shows strongly the value, even in forming a classification, of the opinion of Dr. Baillie, that an examination of diseased changes should lead to an attentive observation of symptoms. But, although we are inclined to think that, in a practical point of view, the more limited application of hypertrophy is the better one, we see at the same time the extreme difficulty of classifying satisfactorily these intermediate cases, particularly in the present imperfect state of our knowledge of pathological anatomy.

There are many instances of hypertrophy in which the operation of any of these causes cannot be satisfactorily established. Such are "accumulation of fat in the form of tumour under the skin, around the kidneys and basis of the heart, and in other parts of the body; the anormal development of the hair of the head and pubis, the epidermis and nails. The *Cutis pendula, Dermatolysis,* &c. of authors is a striking example of hypertrophy of the skin, the cause of which is unknown. It is characterized not so much by an increase of thickness as by great extension of the skin, which is thrown into folds, sometimes several inches in breadth, which, when grouped together, form large pendulous masses: it occurs chiefly in the skin of the upper part of the face, chest, and abdomen. We give an instance of this affection in the Third Part of the present Number, which is still more curious from being hereditary. Of this kind is also the following remarkable instance of dilatation of the lymphatics: it is illustrated by an admirable drawing.

"My friend, M. Amussat, of Paris, was called to a young man, (æt. 26,) who the day before had been seized with severe pain in the abdomen, followed by frequent vomiting. These symptoms, and the presence of two swellings, one in each groin, nearly as large as an orange, left no doubt that the patient was labouring under the effects of strangulated hernia; but the state of prostration was such, that reduction by an operation was not attempted. On examining the patient after death, the only remarkable circumstance observed was enormous dilatation of the lymphatics from both groins upwards, including the thoracic duct. The two swellings situated in the groins, and which, at an early age of the patient, had been treated as a case of *double herniæ*, (for we afterwards learned that he had worn a double truss from his boyhood,) were found to be produced by great dilatation of the lymphatics of the inguinal glands. When cut into, instead of having a compact structure, they presented the appearance of a coarse sponge, from the size of all these vessels being increased; the most of them presenting from one to three lines in diameter. All the lymphatics of the pelvic and lumbar regions presented the same alteration in a still more remarkable degree. None of them were less than two, many of them from three to four lines; and the thoracic duct was from six to eight lines in diameter. As no obstacle was found in the course or at the termination of the thoracic duct, to account for the dilatation of the lymphatics in this singular case, and as these vessels had undergone no other perceptible change, I am disposed to consider it as an example of malformation of these vessels, more especially when these facts are connected with the circumstance furnished by the history of the case,—viz. the existence, at an early period of life, of the enlarged state of the inguinal glands." (Fasc. ix.)

What has been called concentric hypertrophy is an example of the necessity of our having a previous knowledge of the causes in which it originates in individual cases, in order to form a correct estimate of its nature. This depends on arrested or imperfect development, in consequence of which the organ retains after birth the proportions of bulk which were necessary to carry on the fœtal circulation.

"The thickness of the walls, compared with the capacity of the ventricles of the heart in the fœtus, is nearly twice that of the walls compared with the capacity of this organ in the adult. This relative disproportion between the walls and cavities of the fœtal heart is conspicuous in infancy and childhood; but, during the regular development of the body, it gradually merges into those proportions of capacity and bulk which are regarded as indicating, in these respects, the normal state of the heart. If, therefore, the changes which should take place in the regular development of the heart after birth are prevented, it is obvious that the permanence of the state of the fœtal heart which we have described must, under new and so very different conditions of life, constitute a pathological state, a state of concentric hypertrophy, and give rise at an early period to those derangements of function which follow as the consequences of this disease; and that such is the origin of some cases of this form of hypertrophy is proved by a careful examination of their early history, and of the organ itself after death. . . . The persistance after death of the fœtal state of the liver, as to the greater size of the left lobe, is occasionally met with, and might possibly interfere with the process of digestion; and the thymus gland has been found to retain for a considerable time after birth its original dimensions, and to give rise to distressing and even fatal consequences." (Fasc. ix.)

Dr. Carswell's observations on the formation of circumscribed aneurism of the heart, besides their originality, possess considerable interest at the present time, as attention is becoming generally directed to diseases of the internal linings of the cavities coincident with pericarditis.* He is satisfied that the formation of circumscribed aneurism of the heart is a process similar to that of aneurism of vessels. The first perceptible lesion is in the serous membrane lining the internal surface of the ventricle, which within a circumscribed space is of a pale straw colour, opaque, and closely united to the cellular tissue beneath it, which is thickened: occupying the situation of these changes are one or more depressions, cavities, or sacs, lined by serous membrane, whose power of resistance being impaired by disease, appears as if it had been pushed outwards by the contraction of the heart on its contents, carrying before it and producing atrophy of the muscular coat; the only change which it suffers. In the cases where there had been uniform dilatation of the ventricle, the opposite sides of the pericardium were intimately united by firm cellular tissue, the consequence of a previous attack of pericarditis. The same changes were observed in the serous lining and sub-cellular tissue; and there was also a greater or less quantity of cellulo-fibrous tissue occupying the situation of the muscular substance of the ventricle. This does not originate in coagulable lymph; for Dr. C. has distinctly traced it to the separation of fibrine from blood forced into the substance of the heart, in consequence of softening and rupture of some of its muscular fibres.

Dr. Carswell concludes this fasciculus with a general sketch of the physical characters of hypertrophy.

Atrophy is the subject of the tenth fasciculus, and is regarded as depending on an opposite condition to that of hypertrophy, or a diminished exercise of the nutritive function.

The changes gradually produced in the various tissues of the body by the progress of time are ably and clearly explained. The

* See our Review of Bouillaud's work in the present Number.

local forms of atrophy, which in general are permanent pathological conditions, are referred to three heads.

1. Atrophy from a diminished supply of blood. This is one of the most obvious causes, and is seen when an artery supplying an organ is obstructed, and its office is not supplied by the collateral circulation. Thus, atrophy of the brain sometimes follows ossification of the carotid and vertebral arteries within the cranium; and the same change is not unfrequently seen in the inferior extremities from this and other diseased states which interrupt the circulation of the blood through the larger arteries of these parts. Compression of the capillary vessels is a frequent cause of this form of atrophy.

"The stinted growth of some parts of the body, as the feet in some women, and the diminished dimensions of the inferior diameter of the cavity of the thorax in others, is partly produced by compression, so applied as to reduce the quantity of blood usually transmitted through the capillary vessels of these parts; although in both cases the comparative state of inactivity in which the muscles are placed by this cause contributes greatly to the same effect. The liver, in some old women who employ a string or cord to fix their petticoats around the waist, presents on its convex surface a transverse fissure, which is sometimes from half an inch to an inch in depth." (Fasc. x.)

Compression by accidental products is another cause of atrophy. The formation of serous cysts in the kidneys is the most frequent cause of atrophy of these organs, which is often followed by partial or total suppression of urine.

In the majority of cases of insanity which terminate in general paralysis, there is a greater or less accumulation of serosity in the cavity of the arachnoid and in the pia mater between the convolutions of the brain with various degrees of atrophy of that organ, more especially of the grey substance. The presence and extent of the effusion are readily recognized by the colour of the membranes covering it, which present the appearance of irregular, milk-white patches; they are thickened, and in general united together so as to form a sac for the effusion. On removing the membrane, irregular depressions are perceived between the convolutions, which are variously reduced in bulk. It is a matter of doubt whether the atrophy of the brain is produced by chronic irritation, and the serum is effused to supply its place; or whether the atrophy is the consequence of the compression produced by the serum effused by the diseased membranes. The evidence of chronic inflammation of the membranes render the latter supposition the most plausible, but Dr. Carswell believes that it is more than probable that the diseased state of the membranes depends on some change in the functions of the convolutions.

After explaining the mode in which emphysema of the lungs produces atrophy of these organs, Dr. Carswell dwells for some time on an important form of atrophy produced by the compression of a contractile fibrous tissue which is sometimes formed upon the surface of some organs and within others. We shall not regret that our space will not allow us to give more than an outline of Dr. Carswell's remarks, if, by so doing, we shall direct attention to the fasciculus itself, which is full of valuable matter. In chronic pleurisy the lung is at first compressed by effused fluids; and, if these are removed, it regains its size: but it frequently happens that the coagulable lymph which the effusion contained becomes organized, and forms a strong fibrous membrane over the lung, retaining it in its position; and, in proportion as it acquires its fibrous character, it contracts and still further compresses and diminishes the lung; in an extreme case, the greater number of the bronchi and blood-vessels are obliterated and diminished, the pulmonary is replaced by cellular tissue, and the whole enclosed in a dense fibrous capsule, from an eighth to a quarter of an inch thick. That this atrophy is the effect of compression by the fibrous tissue is proved by one lobe occasionally becoming atrophied in this manner, or appearing as if strangulated by a zone of this tissue. The abdominal viscera are sometimes similarly compressed by a fibrous membrane formed on the peritoneal covering of the viscera, after chronic peritonitis.

"The intestines are often found much reduced in capacity, grouped together into a comparatively small mass, and firmly fixed down to the spine; the stomach and urinary bladder are flattened and compressed against the surfaces with which they are in contact; the gall-bladder is contracted and nearly empty; even the liver and kidneys in some cases appear to have undergone a diminution of bulk, and the spleen in general is very small." (Fasc. x.)

Dr. Carswell attributes to the same cause contraction of the œsophagus succeeding ulceration, and stricture of the intestines following the ulceration of Peyer's glands, in both of which instances the muscular coat is partially involved. He makes no mention of the contractions of the skin from burns, which are certainly analogous to contraction after ulcers of mucous membranes. In these cases a new membrane, having strongly contractile powers, is formed.

That peculiar disease of the liver called by Laennec "Cirrhose" is considered by Dr. C. to be an example of atrophy produced by the compression of a fibrous membrane. In this disease the liver is sometimes reduced to one fourth its natural bulk, and its density is increased;

"It appears shrunk, and has an irregularly rounded form, particularly at its edges; and the whole of its external surface is raised into round, flat projections, varying from the size of a hempseed to that of a pea, or even a small cherry. Examined more narrowly, the round flat projections are found to be composed of several smaller ones, and these, again, of the individual lobules of the liver; so that the larger projections are formed of aggregated groups of lobules, each separated the one from the other by cellulo-fibrous or fibrous tissue. . . . When the structure has been exposed by incision, the cut surface presents the same tuberiform arrangement seen on the external surface, the lobules being grouped into smaller or larger masses, mostly of a round, ovoid, or pyriform shape. The cellulo-fibrous or fibrous tissue now forms a conspicuous feature in the disease, both on account of its greater quantity compared with that of the lobular structure of the liver, and the contrast of its white or grey colour with the rusty, yellowish, or greenish-brown colour of the lobules." (Fasc. x.)

To those who are acquainted with Mr. Kiernan's description of Glisson's capsule, which he has traced and minutely described as forming a sheath to the vessels and investing each of the lobules, the explanation given by Dr. Carswell of this disease will be both satisfactory and easily understood. He supposes that it is converted into a fibrous tissue which produces atrophy of the lobules by compressing them. The tuberiform appearance of the external surface is owing to the fibrous tissue which is attached to the peritoneal covering, pulling that membrane inwards all around the groups of lobules. Laennec attributed the disease to the deposition of a new tissue of a rust-brown colour, on which account he called it "Cirrhose." If, however, Dr. Carswell is correct, instead of the formation of a new tissue, it consists of an opposite condition,—a state of atrophy of the lobules. The plates give a clear idea of this disease, which has been much mystified by Laennec's name, and by its being sometimes called tubercular liver; a term which Dr. Carswell has very properly changed to "tuberiform." We believe it is this state of liver which is often called "gin liver," in this country, and "whiskey liver" in Ireland, from the belief that it is produced by distilled spirits. The nature of this affection, says Dr. C., explains the occurrence of ascites, its constant attendant. At the commencement of the disease, when the quantity of fibrous tissue is small, the effusion is inconsiderable; whereas, at a later period, when this tissue has increased, and by its contraction opposed a greater obstacle to the return of the blood from the viscera, it is often very great.

2. The second local cause of atrophy is a diminished supply of nervous influence. It is difficult to say, in paralysis, whether atrophy is owing to this cause or inactivity of the part; but, even in this case, the atrophy is generally greater than could be explained by mere diminished muscular action. It is still more satisfactorily shown in the great extent of wasting of a limb after injuries which interrupt the function of the principal nerve. Atrophy of the arm from compression of the brachial plexus in luxation of the head of the humerus is an example. Dr. Carswell also thinks that the atrophy which accompanies painter's colic, and some of the worst forms of what is called dyspepsia, in highly nervous, hysterical, and hypochondriacal individuals, is, at least in part, the consequence of the same morbid condition of the nerves which gives rise to the diseases, and which, acting on the capillaries, either retards the circulation of blood through them, or prevents it from undergoing those changes necessary to nutrition. With these views of Dr. Carswell's we are disposed to agree, as they explain on correct physiological principles the possibility of atrophy without any appreciable organic disease of the chylopoietic organs. The temporary atrophy, which is not an uncommon symptom among hypochondriacs, who often get thin and stout in the course of a few days, or even less, is thus explained by a temporary disturbance of the functions of the nervous system. Some cases have been recently reported in which dissection proved that atrophy may alone depend on disease of the spinal marrow. Whytt, who described a nervous atrophy as a symptom of hypochondriasis, attributed the wasting frequently to an "unnatural or morbid state of the nerves of the stomach and intestines." His reasons for this opinion are so judicious that we shall make no apology for giving them.

"The influence of the stomach in the animal economy is greater than is perhaps generally imagined. It not only contributes to the digestion of the aliment, but the whole system is either invigorated or affected with a languor, according to the different disposition of its nerves. By proper food, the nerves of the stomach are gratefully stimulated, and the whole body is thus enlivened and strengthened; so that, besides its use for nutrition, food in the stomach becomes, on account of its *stimulus*, altogether necessary, in some delicate nervous people, for keeping up the strength of the body and the due exercise of all its functions: and hence it is that such persons become often faintish as soon as the greatest part of the food has passed into the intestines; that strong broths, though they may afford as much or more nourishment than some kinds of solid meat, yet do not satisfy the stomach, at least for any considerable time, or enable us to endure much labour; and that, according to the different dispositions of the nerves of the stomach, different stimulants are most grateful to it, and most invigorating to the body. We know that an unnatural state of the nerves of the stomach may either produce a craving or an aversion to food; that low spirits and melancholy often proceed from that cause; nor is it to be doubted that, when the nerves of the stomach are, from certain causes, affected in a manner somewhat different, an indifference for food, a weak digestion, a languor and coldness, a slow pulse and wasting, may be the consequences. The morbid affection of the nerves of the stomach, by sympathy, impairs the vigour and energy of the whole system: whence the motion of the heart and the circulation of the blood will become slower and more languid, the body will be deprived of its natural heat, and be affected with a general weakness. The patient decays daily, though exhausted by no excessive evacuations, because his food is not converted into good chyle; and the nutritious fluid in the blood either does not possess its usual properties, or, on account of the languid manner in which all the operations of the body go on, is not applied to the several parts in such a way as to repair the waste they daily suffer. Further, the watching or want of refreshing rest, and low spirits or melancholy, which generally accompany this disease, may contribute to prevent the proper nutrition of the body."*

3. The third local cause of atrophy is the diminished exercise of the function of an organ. This is most striking in the muscles of voluntary motion, but those of involuntary motion are also subject to it. Thus in artificial anus, the muscular coat of the intestines below the opening is reduced to a thin transparent membrane. Laennec relates the case of a woman of fifty, who died of cholera two years after she was cured of hypertrophy of the heart by the treatment of Valsalva, whose heart was found of the size of that of a child twelve years old, looking externally like a withered apple, the wrinkles running longitudinally. Compression of the larger bronchial tubes by enlarged tubercular glands may produce atrophy of the corresponding lung. Dr. Carswell has given a drawing which beautifully illustrates this. A mass of tuberculated glands had compressed the left division of the trachea of a monkey, and, in consequence, the left lung was reduced to nearly one-fourth the size of the other. There was no effusion in the chest, but the parietes of the left side had become depressed as in chronic pleurisy. Atrophy of the mammæ in females who have ceased to menstruate, and the imperfect state of the testicles of those who have spent their youth "under the humiliating and blighting influence of certain monastic vows," come under this head. In this classification of causes, Dr. Carswell has followed Andral. The French pathologist also enumerates "irritation," which we think Dr. Carswell has acted judiciously in omitting, as atrophy from this cause evidently depends on a diseased action, and not merely on a decrease of the healthy nutritive function. The symptoms prove this; for, in atrophy of the breast which sometimes succeeds enlargement of the organ, there is often much local uneasiness.

We cannot sufficiently recommend this work to the attention of our readers, more especially to those among our younger brethren, who, having left the schools, are just entering on the practice, and (as we would fain hope) the *practical study*, of their profession. In Dr. Carswell they will find an enlightened guide to the exact knowledge of organic diseases, and one whom they may trust with the most undoubting confidence. One of the grand charms to us in the writings of Dr. C. is, that they are evidently the productions of a thoroughly *honest* enquirer: he may be wrong, and no doubt he occasionally is wrong; but the reader may always feel assured that the author believes himself to be right, and that he has taken all possible pains to be so.

The illustrations both of Hypertrophy and Atrophy are numerous, and are, like those which preceded them, no less admirable for their pathological truth than for their pictorial beauty.

* The works of R. Whytt, M.D. Quarto. P. 600.

